# Short-Term Associations between Size-Fractioned Particles and Cardiopulmonary Function in COPD Patients: A Panel Study in Shanghai, China, during 2014–2021

**DOI:** 10.3390/ijerph191912473

**Published:** 2022-09-30

**Authors:** Lu Zhou, Yingmin Tao, Xiaozhen Su, Xiyin Chen, Liang Li, Qingyan Fu, Juan Xie, Renjie Chen

**Affiliations:** 1 Department of Environmental Health, School of Public Health, Fudan University, Shanghai 200032, China; 2 Division of General Practice, The Fifth People’s Hospital of Shanghai, Fudan University, Shanghai 200240, China; 3 Center of Community-Based Health Research, Fudan University, Shanghai 200240, China; 4 Department of International Health, Johns Hopkins Bloomberg School of Public Health, Baltimore, MD 21205, USA; 5 Shanghai Environmental Monitoring Center, Shanghai 200235, China

**Keywords:** size-fractioned particles, panel study, COPD, cardiopulmonary function

## Abstract

It remains unknown which size fractions dominate the adverse cardiopulmonary effects of particulate matter (PM). Therefore, this study aimed to explore the differential associations between size-fractioned particle number concentrations (PNCs) and cardiopulmonary function measures, including the forced expiratory volume in one second (FEV_1_), the forced vital capacity (FVC), and the left ventricular ejection fraction (LVEF). We conducted a panel study among 211 patients with chronic obstructive pulmonary disease (COPD) in Shanghai, China, between January 2014 and December 2021. We applied linear mixed-effect models to determine the associations between cardiopulmonary function measures and PNCs ranging from 0.01 to 10 μm in diameter. Generally, only particles <1 μm showed significant associations, i.e., ultrafine particles (UFPs, <0.1 μm) for FVC and particles ranging from 0.1 to 1 µm for FEV_1_ and LVEF. An interquartile range (IQR) increment in UFP was associated with decreases of 78.4 mL in FVC. PNC_0.1–0.3_ and PNC_0.3–1_ corresponded to the strongest effects on FEV_1_ (119.5 mL) and LVEF (1.5%) per IQR increment. Particles <1 µm might dominate the cardiopulmonary toxicity of PM, but UFPs might not always have the strongest effect. Tailored regulations towards particles <1 µm should be intensified to reduce PM pollution and protect vulnerable populations.

## 1. Introduction

Chronic obstructive pulmonary disease (COPD) is of growing global concern owing to its increasing prevalence, mortality, and substantial financial burden worldwide [1,2,3]. As the sixth leading cause of death, COPD was estimated to cause 3.28 million deaths worldwide in 2019, most of which occurred in low- and middle-income countries [2]. Notably, cardiopulmonary dysfunction is a frequent physical condition seen among patients with COPD [4] and is closely related to the prognosis of survival and the quality of life [5,6]. Therefore, identifying the potential risk factors contributing to cardiopulmonary dysfunction among COPD patients is of great significance. In general, the evolution of the research field of COPD has long been driven by scientific problems and led by high-income countries [7]. The COVID-19 global pandemic has raised unprecedented concern worldwide, posing a dramatic impact on public health, particularly for the elderly with chronic diseases. However, the impact of air pollution cannot be overemphasized in the COVID-19 era [3]. It has been reported that air pollutants could perturb the immune system by affecting various immune cell types and inducing immune responses, leading to COPD exacerbation and disease progression for the susceptible subgroup [8,9,10].

Mounting epidemiological studies have linked exposure to particulate matter (PM) air pollution to adverse cardiopulmonary health outcomes [3,11,12,13]. Previous studies have also investigated the short-term associations between PM and cardiopulmonary function, but the current evidence is still uncertain [14,15,16]. Moreover, although a few studies have explored the differentiated effects of size-fractioned PM on cardiopulmonary health [17,18,19] and have found that ultrafine particles (UFPs) might have potentially enhanced toxicity compared with larger particles [20], the related epidemiological evidence is still inconsistent and limited [21]. For instance, several investigations suggested a significant association between UFP exposure and lung function [22,23], whereas others reported a weak or no association [24,25]. Moreover, previous studies have assessed the short-term effects of UFPs on cardiac autonomic function and blood pressure [17,25,26], but no study evaluated the systolic cardiac function. Additionally, there remain considerable knowledge gaps concerning the size-dependent effects of PM on cardiopulmonary function across the entire size spectrum [17,27].

In recent years, China has been plagued with PM air pollution [11]. Additionally, COPD is highly prevalent in China, with an overall prevalence of 8.6% in adults, equivalent to over 100 million COPD patients [28]. Particularly, patients with COPD are more susceptible to cardiopulmonary impairment induced by PM air pollution [8]. Therefore, this study aimed to explore the associations between short-term exposure to the size-fractioned PM and cardiopulmonary function among a panel of COPD patients in Shanghai, China.

## 2. Materials and Methods

### 2.1. Sample and Data

This panel study was conducted between January 2014 and December 2021 among COPD patients. Based on the medical records of the Shanghai Fifth People’s Hospital, we included the participants who were adults ≥40 years of age and permanent residents of Shanghai with at least two recorded cardiopulmonary function measurements during the study period. All the participants had a physician-confirmed diagnosis of stable COPD (without recent exacerbation) according to the Global Initiative for Chronic Obstructive Lung Disease (GOLD) criteria [29]. The exclusion criteria for the study included a history of chest surgery, lung cancer, pneumoconiosis, or occupational exposure to fumes/dust. Moreover, we excluded the participants who experienced an acute exacerbation of COPD during the 3 months prior to the clinic visits. We also collected individual information, including age, sex, body mass index (BMI), smoking status, medication use, and exacerbation history. The participants for whom there were missing data on the date of clinic visits and individual information were also excluded from the analysis. This study was approved by the Institutional Review Board of Shanghai Fifth People’s Hospital (NO. 2020-3), with a waiver of informed consent.

### 2.2. Measures of Variables

Lung function parameters were assessed via spirometry (Quark PFT3, COSMED, Rome, Italy), including the forced expiratory volume in one second (FEV_1_) and forced vital capacity (FVC). These parameters have been extensively used and verified in previous studies among pulmonary patients [30,31]. All lung function tests were performed under the guidance of well-trained technicians following recommended procedures [32]. For cardiac function, a standard four-dimensional Doppler echocardiography (GE Vivid E95 Ultrasound System, GE Healthcare, Horten, Norway) was performed by experienced operators to evaluate the left ventricular ejection fraction (LVEF). All the measurements were obtained under the recommendations of the American Society of Echocardiography [33]. LVEF is a widely used index to assess the left ventricular systolic function, and LVEF < 50% indicates systolic dysfunction [34].

The real-time particle number concentrations (PNCs) of size-fractioned PM were collected from a nearby monitoring supersite, which was the only supersite operated in Shanghai throughout the study period. Located in an urban area, this fixed-site station has been shown to well reflect the background levels of PNCs previously [17]. The real-time PNCs across various sizes were measured using either a scanning mobility particle sizer (SMPS) or an aerodynamic particle sizer (APS) (TSI Corporation, Upper Marlboro, MD, USA) through multiple size channels. Specifically, SMPS measures the size distribution of aerosols ranging from 0.01 to 0.75 μm, and APS measures the size distribution of aerosols ranging from 0.5 to 10 μm. Then, the PNCs were directly obtained from the measurements using SMPS for particles of sizes ranging from 0.01 to 0.75 μm. For the PNCs of particles ranging from 0.75 to 10 μm, we applied a correction factor to calculate the SMPS-APS merged data, given the inherent difference between the measures from SMPS and APS [35]. The exponential curves of the PNCs were firstly fitted for SMPS and APS against their overlapped ranges by the size distribution (0.50–0.75 μm) to obtain the size correction factor. Then, we calculated the ratio of size ranges at the same concentrations from APS and SMPS, and finally determined this factor as 1.2248. In the present study, we obtained the daily average PNCs for size-fractioned PM segregated into five size ranges, including 0.01–0.1 µm (PNC_0.01–0.1_, UFPs), 0.1–0.3 µm (PNC_0.1–0.3_), 0.3–1 µm (PNC_0.3–1_), 1–2.5 µm (PNC_1–2.5_), and 2.5–10 µm (PNC_2.5–10_).

To control for the confounding effects of the criteria air pollutants, we collected the data on the daily mean concentrations of particulate matter less than 2.5 μm in aerodynamic diameter (PM_2.5_) and gaseous air pollutants, including ozone (O_3_, maximum 8 h mean), nitrogen dioxide (NO_2_), sulfur dioxide (SO_2_), and carbon monoxide (CO). These air pollution data were obtained from the nearest monitor site around the hospital (approximately 1.0 km away). For adjustment to weather conditions, we also collected the daily meteorological data (i.e., the mean temperature, relative humidity, and wind speed) from a nearby meteorological station operated by the Shanghai Meteorological Bureau.

### 2.3. Models and Data Analysis Procedure

Firstly, environmental exposure and the health outcome data were linked by the date of each health measurement. Linear mixed-effect (LME) models were applied to examine the associations between size-fractioned PM and the measures of cardiopulmonary function (i.e., FEV_1_, FVC, and LVEF). This model accounts for the within-participant correlations of repeated measurements and adjusts for within-participant time-invariant covariates. In each LME model, we included an identity number for each participant as the random-effect intercept to account for the within-participant variations and incorporated the following covariates to control for the potential confounding effects, including individual characteristics (e.g., age, sex, BMI, smoking status, and medication use); a natural cubic spline smooth function of calendar day with 3 degrees of freedom and the indicator variables of the day of the week to control for time trends; the natural cubic spline smooth functions of the daily average temperature and relative humidity with 3 degrees of freedom; and a natural cubic spline smooth function of the 2-day moving average of the wind speed (the current day and the previous day before the clinic visit) with 3 degrees of freedom. Given the correlations between PNCs and PM, we also included the 2-day moving average PM_2.5_ concentrations in the models as a surrogate for the adjustment of PM [11].

Given that the health effects of air pollution may last for more than one day, we examined the temporal pattern for the associations between the PNCs of size-fractioned PM and cardiopulmonary function measures. According to previous studies and our prior analytical results [36,37], we fitted the models using various lag structures, including the single-day lag (lag 0 d) and the multi-day lag (lag 01–03 d). For example, lag 0 d corresponded to the exposure on a concurrent day, whereas lag 01 d corresponded to the exposure on the moving average of the current day and one day prior to the clinical visit. The optimal lag interval was determined based on the Akaike Information Criterion (AIC) values. In addition, we performed a sensitivity analysis to evaluate the robustness of the results by fitting the multi-pollutant models with adjustment of four gaseous air pollutants (i.e., NO_2_, SO_2_, O_3_, and CO) at lag 01 d.

All analyses were performed in R (Version 4.0.3, R Foundation for Statistical Computing) using the “*lme4*” package. The effect estimates for size-fractioned PM were presented as mean changes and their 95% confidence intervals (CIs) associated with an interquartile range (IQR) increase in PNCs across various cardiopulmonary function measures.

## 3. Results

### 3.1. Descriptive Statistics

The summary statistics of the participants are presented in Table 1. A total of 211 participants (171 males and 40 females) with an average age of 73.6 years were included in this study. Finally, 482 medical records of repeated clinic visits with eligible measurements were collected during the study period. A total of 171 patients had 2 eligible repeated records, and 40 participants had 3 or more eligible records. The average duration between the clinic visits was 324 days. Among these participants, 52 participants were current smokers. The mean FEV_1_, FVC, and LVEF were 1094.3 mL, 722.4 mL, and 64.5%, respectively.

Table 2 summarizes the statistics of environmental variables on the current day of clinic visits during the study period. From 2014 to 2021, the PNCs of size-fractioned PM were distributed disproportionately across various size ranges, with much more number concentrations in the particles ranging from 0.01 to 0.3 μm. Additionally, the daily mean concentrations of the five criteria air pollutants were 37.7 μg/m^3^ for PM_2.5_, 44.0 μg/m^3^ for NO_2_, 10.4 μg/m^3^ for SO_2_, 96.1 μg/m^3^ for O_3_ (maximum 8 h mean), and 0.78 mg/m^3^ for CO, respectively. As shown in Table S1, we found weak-to-moderate correlations between PNCs and air pollutants or weather conditions. The PNCs of particles with a size of 0.1–1 μm were more strongly correlated with air pollutants than in other sizes. The correlations of PNCs with PM_2.5_ ranged from −0.06 (PNC_0.01–0.1_) to 0.66 (PNC_0.1–0.3_). Moreover, all size-fractioned PM showed weak correlations with weather conditions. Notably, the wind speed was negatively associated with the PNCs of the particles, with the correlations ranging from −0.25 to −0.04.

### 3.2. Regression Results

**Figure 1:**
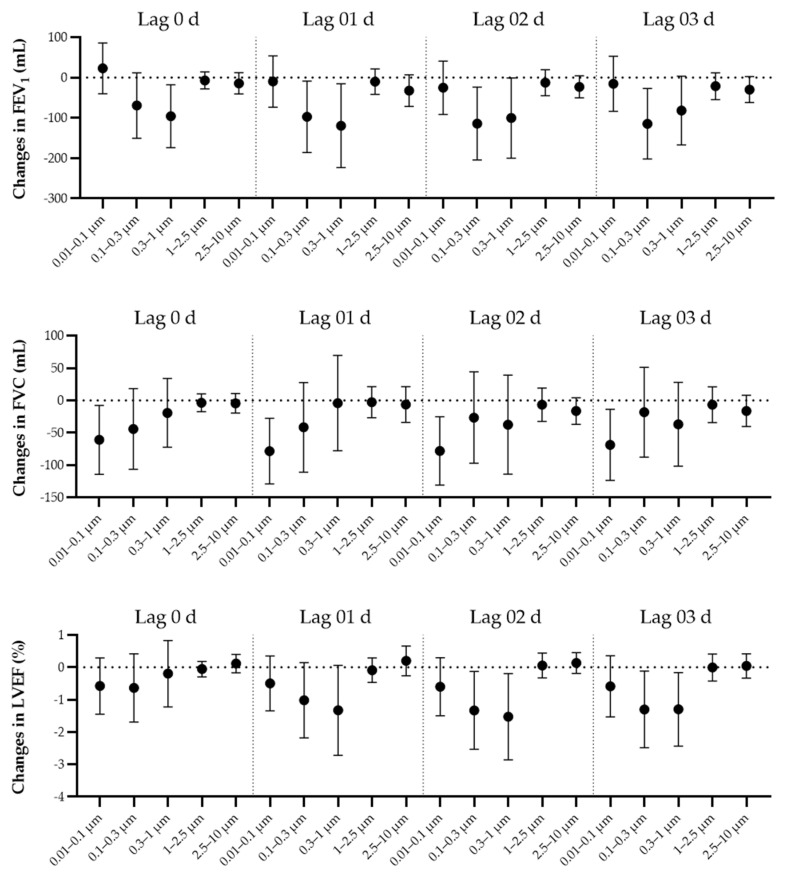
Changes (mean and 95% confidence intervals) in cardiopulmonary function measures associated with an interquartile range increase in size-fractioned PNC using different lag days, adjusted for PM_2.5_. Abbreviations as in Table 1 and Table 2.

Our findings showed negative associations between size-fractioned PM and cardiopulmonary function. Figure 1 shows the associations between the PNCs of PM and cardiopulmonary function measures by size and temporal characteristics. Generally, the magnitude of these associations increased from lag 0 d to lag 01 d and then attenuated for FEV_1_ and FVC, while the magnitudes of these associations increased up to lag 02 d for LVEF. Therefore, we selected lag 01 d and 02 d to report the main results for the measures of lung and cardiac function, respectively. Additionally, we did not observe significant associations between all the measures and particles of size >1 μm across various lag days, which were all towards the null across all the measures.

For lung function, the particles of size <1 μm might have potential toxicity, with reduced FEV_1_ and FVC. However, the extent to which these particles affected lung function varied by size fraction and measure. For FEV_1_, the magnitude of the associations strengthened when the particle sizes were <1 μm, but we only observed significant associations when the particle sizes were between 0.1 and 1 μm. For example, an IQR increase in PNC_0.1–0.3_ and PNC_0.3–1_ at lag 01 d was associated with decreases of 97.5 mL (95%CI: 8.9 mL, 186.1 mL) and 119.5 mL (95%CI: 15.7 mL, 232.2 mL) in FEV_1_ (Table 3). Moreover, the effect size of UFPs became smaller and insignificant, with a decrease of 9.9 mL (95%CI: −53.7 mL, 73.5 mL) in FEV_1_ associated with each IQR increase in UFPs. Conversely, we observed the strongest and most significant association in UFPs for FVC, and the associations became weaker and lost significance when the particle size increased. Correspondingly, an IQR increase in PNC_0.01–0.1_ at lag 01 d was associated with a decrease of 78.4 mL (27.7 mL, 129.1 mL) in FVC (Table 3). Additionally, an IQR increase in PNC_0.1–0.3_, PNC_0.3–1_, PNC_1–2.5_, and PNC_2.5–10_ at lag 01 d was associated with decreases of 41.6 mL, 4.1 mL, 2.7 mL and 6.4 mL in FVC.

For cardiac function, the particles of size <1 μm were adversely associated with LVEF, and the associations became stronger with the particle size. We observed significant associations of PNC_0.1–0.3_ and PNC_0.3–1_ for LVEF, and the increment of each IQR in them (lag 02 d) was associated with decreases of 1.3% (95%CI: 0.1%, 2.5%) and 1.5% (95%CI: 0.2%, 2.9%) in LVEF, respectively (Table 3). UFP exposure was not significantly associated with changes in LVEF, and the corresponding effect estimate was 0.6% (95%CI: −0.3%, 1.5%).

**Figure 2:**
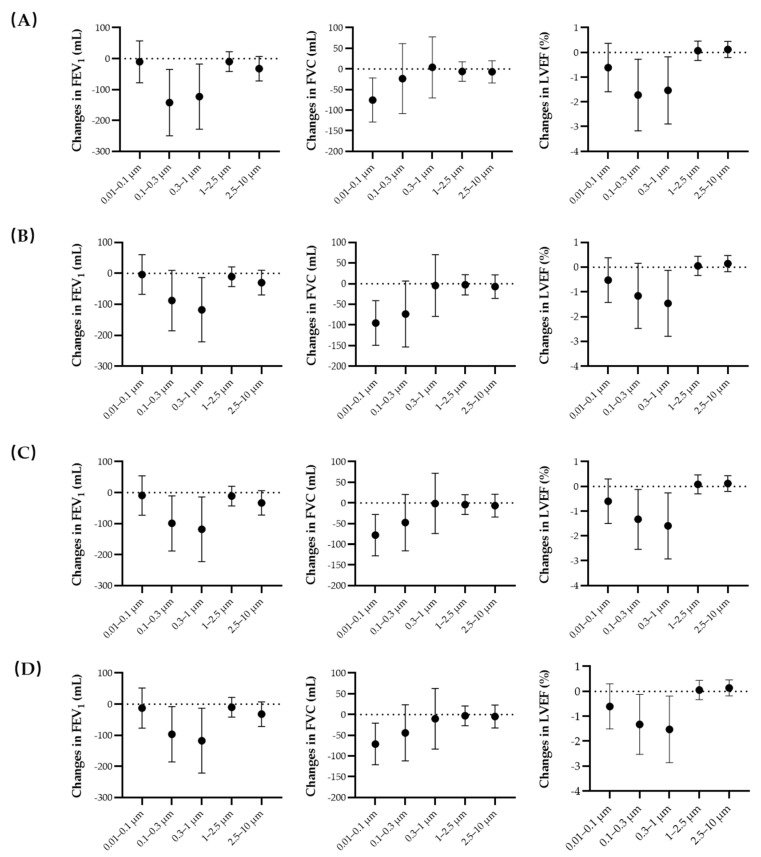
Changes (mean and 95% confidence intervals) in cardiopulmonary function measures associated with an interquartile range increase in size-fractioned PNC, adjusted for PM_2.5_ and (**A**) NO_2_, (**B**) SO_2_, (**C**) O_3_, (**D**) CO. Abbreviations as in Table 1 and Table 2.

After adjustment for gaseous air pollutants, the associations between PNCs and cardiopulmonary function measures remained robust, although the magnitude differed in various models (Figure 2). As shown in Table S2, an IQR increase in PNC_0.3–1_ at lag 01 d was associated with decreases in FEV_1_ ranging from 117.5 mL to 123.2 mL across the models (*p* < 0.05). For FVC, an IQR increase in UFPs was significantly associated with decreases in FVC after adjusting for gaseous air pollutants, with estimates fluctuating between 71.2 mL and 95.1 mL. Additionally, the results of the sensitivity analysis on LVEF were robust. An IQR increase in PNC_0.3–1_ at lag 02 d was associated with decreases of 1.5% or 1.6% in LVEF.

## 4. Discussion

To our knowledge, this is one of the limited panel studies regarding the acute effects of size-fractioned PM on cardiopulmonary function among COPD patients. Our findings indicated that the smaller particles with size <1 µm had adverse effects on lung function, and the particles of sizes between 0.1 and 1 µm might dominate the toxicity of PM for cardiac function. These findings were fairly robust after adjustment for the PM_2.5_ total mass and gaseous air pollutants. This study added new evidence to the limited investigation examining the deleterious effects of size-fractioned PM on cardiopulmonary health in COPD patients.

The lagged effects of PM exposure on health outcomes have been widely established, but the findings on the lag pattern of size-fractioned PM were still limited and inconsistent. In the present study, we found the strongest associations between PNCs and cardiopulmonary function measures at lag 01 or lag 02 d, indicating the acute effects of PM across various ranges of size. Similarly, previous studies also suggested the short-term effects of size-fractioned PM on adverse outcomes [38,39,40]. For instance, a longitudinal panel study in Shanghai found the strongest effects on inflammatory biomarkers occurred within 12–24 h after exposure to size-fractioned PM [38]. Inversely, it has been reported that exposure to PM might induce more lasting impacts on airway inflammation and respiratory symptoms among COPD patients [41]. Therefore, further studies were warranted to elucidate the lagged effects of particles over a wide range of sizes and address this issue.

There was inconsistency in epidemiological evidence on the associations between PM and lung function. In line with previous studies, we found a negative association between PM and lung function in COPD patients [31,39,40,42]. For instance, a longitudinal panel study in Beijing suggested that each 10 μg/m^3^ increment in PM_2.5_ was associated with decreases of 0.014 L in FEV_1_ and 0.025 L in FVC [39]. Similarly, another panel study also observed lung function decline in response to PM exposure, including PM_1.0_, PM_2.5_, and PM_10_ [40]. Nevertheless, a multi-center cross-sectional study in southern China found a significant association between PM and lung function in the general population rather than in COPD patients [43]. Generally, these investigations predominated in PM_2.5_ and PM_10_, and much less evidence is available on the differential effects of size-fractioned PM on pulmonary health [18,19,30]. To our knowledge, we are one of the first to investigate the associations between lung function and the PNCs of particles across a wide range of sizes among COPD patients. Nonetheless, several studies might directly support our findings [18,44]. For instance, an interventional study showed that reduced FVC was associated with particles of ≤0.02 μm rather than particles of >0.05 μm among healthy volunteers [18]. Moreover, a research study in Shanghai suggested that the particles ranging from 0.25 to 0.5 μm were closely linked with an increased risk of COPD mortality, and the associations became stronger for smaller particles [44]. In this study, an IQR increment in PNC_0.25–0.28_, PNC_0.28–0.3_, PNC_0.3–0.35_, PNC_0.35–0.4_, PNC_0.4–0.45_, and PNC_0.45–0.5_ was associated with increments of 7.51%, 7.22%, 6.95%, 6.26%, 5.24%, and 4.15% in COPD mortality, respectively. Generally, impaired lung function was significantly associated with particles of <1 μm rather than larger particles, although the exact size fractions varied across studies.

Consistent with previous studies, our findings suggested the significant associations between PM and impaired cardiac function, represented by reduced LVEF. LVEF is a widely used clinical indicator for the assessment of left ventricular systolic function and the diagnosis of cardiac dysfunction and heart failure [34]. Our previous study has shown its sensitivity to PM exposure among COPD patients and its promising prospect in environmental epidemiology [31]. To our knowledge, there has been no study investigating the association between LVEF and PM covering the entire size spectrum, which might limit the comparability of our findings. However, previous studies have suggested the cardiovascular effects of size-fractioned PM among healthy and vulnerable populations. For instance, the size-fractioned PM might reduce cardiovascular function, represented by impaired cardiac automatic function (e.g., reduced heart rate variability), increased blood pressure, and a higher level of inflammation, blood coagulation, and vasoconstriction [17,18,37,38,45]. Additionally, exposure to size-fractioned PM was suggested to reduce automatic cardiac function in COPD patients, particularly for the smaller particles [27]. An observational study in Shenyang, China, suggested that particles smaller than 0.65 μm were significantly associated with cardiovascular mortality, particularly for particles smaller than 0.35 μm [36]. Further research is needed to clarify the differential effects of size-fractioned PM on cardiac function using more advanced technology and sensitive detection tools.

Generally, the detrimental effects of PM vary greatly by the size of the particles [36,37,38,44]. Consistent with previous studies, we found that smaller particles (<1 μm) might dominate the deleterious health effects of PM on cardiopulmonary health [1,17,18,36,38,40]. It is quite plausible because smaller particles might be more toxic due to the larger surface area with more absorbed toxins, higher alveolar transport and deposition, and higher number concentration [20]. However, a number of studies suggested weak and insignificant associations between UFP exposure and health outcomes [21]. For example, a Dutch birth cohort study reported no evidence for the effect of UFPs on lung function [24]. Our findings also suggested that UFP exposure might not always have measurable adverse health effects. One possible reason is that there was a larger measurement error in the assessment of UFP exposure due to its high spatial and temporal variability, which might bias the effect estimates of UFPs toward the null [21]. In addition, it may be owing to the characteristics and sources of local PM, the distribution of constituents in PM of various sizes, medication use, and the underlying pathology in COPD patients [31,46]. Nevertheless, more research is needed to verify these speculations and bridge the knowledge gaps.

Our study might have important public health implications. We evaluated particles with a wide range of sizes from 0.01 to 10 μm, which allowed for a comprehensive assessment of the differentiated effects of particles and called for further attention shifts from the PM_2.5_ total mass to particle size. Moreover, after adjusting for weather conditions, including wind speed, we still observed significant associations between the particles of <1 μm and cardiopulmonary function measures. Higher wind speed could facilitate the dispersion and dilution of air pollutants and improve air quality [47], but there is less evidence regarding the complex interaction of air pollution and various meteorological factors such as wind speed in producing health effects [17,31,37]. Therefore, more studies are needed to clarify this issue further. In addition, our findings revealed the vulnerability of patients with COPD under the current air quality standard, which demands targeted disease management and improved air quality to protect public health.

Population vulnerability has long been the focus of public health research, which might be exacerbated by the combination of the COVID-19 pandemic and air pollution. PM is a complex mixture of various toxic components, which could induce oxidative stress and inflammation and trigger adverse health outcomes [31]. In response to PM pollution, the immune system plays a crucial role, but it might respond inadequately under a highly polluted environment, particularly among COPD patients with low oxygen supply and vascular or hemodynamic alterations [8,31]. Furthermore, PM might increase the susceptibility by interacting with viruses and affecting the transmission dynamic of COVID-19 [8]. Overall, the health effects of PM air pollution remain an important research topic, and more comprehensive research is needed in the context of the COVID-19 pandemic [7].

Additionally, several limitations should be noted. First, we collected the PNC data from a fixed-site monitoring station instead of individual-level exposure measurements, which could not account for the spatial variability and resulted in inevitable exposure measurement errors. However, it might not have substantially affected our results because the present study focused on the temporal variability of exposure. Further, we collected the data on criteria air pollutants from another station, which may lead to larger uncertainty for the results from multi-pollutant models. Second, the data in this study were collected from medical records with limited sample size, which might not be representative of COPD patients, and any generalization of our findings should be made with caution. Third, we were unable to control for more individual characteristics in our models because we had limited information on the potential confounders, such as socioeconomic status, occupations, and physical activity. Finally, the toxicity of size-fractioned PM might be closely related to chemical compositions because the particles originating from different sources had complex distributions of PM constituents in specific PM size fractions [48]. Therefore, further studies are needed to evaluate the cardiopulmonary responses over a wide range of PM size fractions and size-specific constituents.

## 5. Conclusions

This study provided valuable evidence for the differential effects of PM size fractions on cardiopulmonary health. Particles of <1 μm might dominate the adverse effects of PM on lung function, and particles with a size between 0.1 and 1 μm might contribute to impaired cardiac function. Additionally, UFP exposure might not always have a measurable adverse impact on cardiopulmonary health.

These conclusions are preliminary, as we only evaluated the effects of size-fractioned PM among COPD patients with small sample size. Multi-center and large-sample investigations are warranted to verify our findings, with detailed consideration for the accurate exposure measurements, sources, and constituents of PM, and the complex interaction of air pollution and meteorological factors. Finally, it would help policymakers to establish a comprehensive policy with social, economic, and environmental sustainability to develop tailored air quality regulations and protect public health with intersectoral collaboration. 

## Figures and Tables

**Table 1:** Basic characteristics of the participants (N = 211).

Item	Measurement
Age, years, mean ± SD	73.6 ± 9.5
Gender, N (%)	
Male	171 (81.0%)
Female	40 (19.0%)
BMI, Kg/m^2^, mean ± SD	22.9 ± 3.6
Smoking status, N (%)	
Never	111 (52.6%)
Former	48 (22.8%)
Current	52 (24.6%)
Medication use, N (%)	171 (81.0%)
Cardiopulmonary function measures, mean ± SD	
FEV_1_, mL	1094.3 ± 556.5
FVC, mL	722.4 ± 689.5
LVEF, %	64.5 ± 5.4

Abbreviations: SD, standard deviation; BMI, body mass index; FEV_1_, forced expiratory volume in 1 s; FVC, forced vital capacity; LVEF, left ventricular ejection fraction.

**Table 2:** Summary statistics of air pollution levels and weather conditions on the current day of clinic visits during study period.

Exposure	Mean	SD	P_25_	P_50_	P_75_	IQR
PNC, particles/cm^3^						
0.01–0.1 μm	4229.8	2310.6	2647.3	3769.1	5212.3	2565.0
0.1–0.3 μm	2424.2	1279.5	1476.0	2182.3	3144.9	1668.9
0.3–1 μm	275.6	206.1	127.9	227.9	362.8	234.9
1–2.5 μm	4.4	12.2	0.0	1.9	3.9	3.9
2.5–10 μm	0.2	0.4	0.0	0.0	0.2	0.2
Air pollutants, μg/m^3^						
PM_2.5_	37.7	27.0	20.8	32.5	50.5	29.7
NO_2_	44.0	19.7	30.0	40.9	54.1	24.1
SO_2_	10.4	7.7	5.7	8.5	12.9	7.2
O_3_ (8 h mean)	96.1	45.9	63.5	88.5	121.6	58.1
CO, mg/m^3^	0.78	0.29	0.59	0.74	0.92	0.33
Weather conditions						
Temperature, °C	17.6	8.5	10.2	18.6	24.5	14.3
Relative humidity, %	73.9	12.7	65.0	75.0	83.0	18.0
Wind Speed, m/s	2.5	0.9	1.9	2.4	3.0	1.1

Abbreviations: PM_2.5_, particulate matter with an aerodynamic diameter ≤ 2.5 μm; PNC, particle number concentrations; O_3_, ozone (8 h mean); NO_2_, nitrogen dioxide; SO_2_, sulfur dioxide; CO, carbon monoxide; SD, standard deviation; P_25_, 25th percentile; P_75_, 75th percentile; IQR, interquartile range. P_50_ denotes median.

**Table 3:** Changes (mean and 95% confidence intervals) in cardiopulmonary function measures associated with an interquartile range increase in size-fractioned PNC, adjusted for PM_2.5_.

	FEV_1_ (mL)	FVC (mL)	LVEF (%)
PNC_0.01–0.1_	−9.9 (−73.5, 53.7)	−78.4 (−129.1, −27.7) *	−0.6 (−1.5, 0.3)
PNC_0.1–0.3_	−97.5 (−186.1, −8.9) *	−41.6 (−110.8, 27.7)	−1.3 (−2.5, −0.1) *
PNC_0.3–1_	−119.5 (−223.2, −15.7) *	−4.1 (−77.8, 69.5)	−1.5 (−2.9, −0.2) *
PNC_1–2.5_	−10.0 (−41.6, 21.5)	−2.7 (−26.6, 21.2)	0.1 (−0.3, 0.4)
PNC_2.5–10_	−32.4 (−71.8, 7.0)	−6.4 (−33.9, 21.2)	0.1 (−0.2, 0.5)

Abbreviations as in Table 1 and Table 2. * *p* < 0.05.

## Data Availability

The datasets generated and/or analyzed during the current study are available from the corresponding author upon reasonable request.

## Supplementary Materials

The following supporting information can be downloaded at: https://www.mdpi.com/article/10.3390/ijerph191912473/s1, Table S1: Pearson correlation coefficients of size-fractioned PNC with criteria air pollutant concentrations and weather conditions; Table S2: Changes (mean and 95% confidence intervals) in cardiopulmonary function measures with an interquartile range increase in size-fractioned PNC, adjusted for PM_2.5_ and (A) NO_2_, (B) SO_2_, (C) O_3_, (D) CO.
