# Supplementary material for: Short-Term Associations between Size-Fractioned Particles and Cardiopulmonary Function in COPD Patients: A Panel Study in Shanghai, China, during 2014–2021
Source: Int J Environ Res Public Health. 2022 Sep 30;19(19):12473. doi: 10.3390/ijerph191912473 (PMC9566564; doi:10.3390/ijerph191912473)
Supplement: Supplementary file 1 [file ijerph-19-12473-s001.zip › ijerph-1892589-supplementary.pdf]

Supplementary Materials

Short-Term Associations between Size-Fractioned Particles and Cardiopulmonary Function in COPD Patients: A Panel Study in Shanghai, China, during 2014–2021

Table S1. Pearson correlation coefficients of size-fractioned PNC with criteria air pollutant concentrations and weather conditions.

	**$PM_{2.5}$**	**SO_2**	**NO_2**	**CO**	**O_3**	**Temperature**	**Relative Humidity**	**Wind Speed**
$PNC_{0.01–0.1}$	−0.06*	0.22*	0.08*	−0.02	−0.05*	0.21*	−0.26*	−0.08*
$PNC_{0.1–0.3}$	0.66*	0.24*	0.66*	0.55*	0.43*	−0.19*	−0.27*	−0.25*
$PNC_{0.3–1}$	0.84*	0.05*	0.60*	0.37*	0.55*	−0.27*	0.06*	−0.23*
$PNC_{1–2.5}$	0.19*	0.13*	0.04	0.05	0.12*	0.14*	0.09*	−0.07*
$PNC_{2.5–10}$	0.27*	0.09*	0.14*	0.29*	0.21*	0.02	−0.04	−0.04

Abbreviations: $PM_{2.5}$, particulate matter with an aerodynamic diameter ≤ 2.5 μm; PNC, particle number concentrations; O_3, Ozone (8 h mean); NO_2, nitrogen dioxide; SO_2, sulfur dioxide; CO, carbon monoxide. * $p < 0.05$.

Table S2. Changes (mean and 95% confidence intervals) in cardiopulmonary function measures associated with an interquartile range increase in size-fractioned PNC, adjusted for $PM_{2.5}$ and (A) NO_2, (B) SO_2, (C) O_3, (D) CO.

	FEV_1 (mL)	FVC (mL)	LVEF (%)
$+NO_2$			
$PNC_{0.01–0.1}$	−10.4 (−78.0, 57.2)	−75.6(−129.1, −22.0) *	−0.6 (−1.6, 0.4)
$PNC_{0.1–0.3}$	−142.2 (−249.8, −34.7) *	−23.7 (−108.7, 61.3)	−1.7 (−3.2, −0.3) *
$PNC_{0.3–1}$	−123.2 (−228.8, −17.6) *	3.8 (−70.2, 77.8)	−1.5 (−2.9, −0.2) *
$PNC_{1–2.5}$	−9.6 (−41.6, 22.3)	−6.2 (−29.8, 17.5)	0.1 (−0.3, 0.5)
$PNC_{2.5–10}$	−32.7 (−72.2, 6.9)	−6.9 (−33.7, 19.8)	0.1 (−0.2, 0.5)
$+SO_2$			
$PNC_{0.01–0.1}$	−3.7 (−67.9, 60.5)	−95.1 (−149.1, −41.2) *	−0.5 (−1.4, 0.4)
$PNC_{0.1–0.3}$	−87.8 (−185.5, 9.8)	−73.3 (−153.1, 6.5)	−1.2 (−2.5, 0.2)
$PNC_{0.3–1}$	−117.5 (−221.4, −13.6) *	−4.5 (−79.3, 70.4)	−1.5 (−2.8, −0.1) *
$PNC_{1–2.5}$	−10.8 (−42.4, 22.7)	−2.8 (−27.2, 21.6)	0.1 (−0.3, 0.4)
$PNC_{2.5–10}$	−29.9 (−69.8, 10.0)	−7.1 (−35.5, 21.4)	0.1 (−0.2, 0.5)
$+O_3$			
$PNC_{0.01–0.1}$	−9.4 (−73.1, 54.3)	−78.0 (−127.9, −28.1) *	−0.6 (−1.5, 0.3)
$PNC_{0.1–0.3}$	−99.3 (−188.2, −10.4) *	−47.6 (−115.8, 20.6)	−1.3 (−2.5, −0.1) *
$PNC_{0.3–1}$	−118.5 (−222.8, −14.3) *	−1.4 (−74.6, 71.8)	−1.6 (−2.9, −0.3) *
$PNC_{1–2.5}$	−11.2 (−43.0, 20.5)	−4.0 (−28.0, 20.0)	0.1 (−0.3, 0.5)
$PNC_{2.5–10}$	−33.2 (−72.8, 6.3)	−6.7 (−34.4, 20.9)	0.1 (−0.2, 0.4)
+CO			
$PNC_{0.01–0.1}$	−12.6 (−77.1, 51.9)	−71.2 (−121.5, −20.9) *	−0.6 (−1.5, 0.3)
$PNC_{0.1–0.3}$	−96.9 (−185.9, −7.9) *	−44.2 (−112.0, 23.6)	−1.3 (−2.5, −0.1) *
$PNC_{0.3–1}$	−117.6 (−221.9, −13.3) *	−10.3 (−83.5, 62.8)	−1.5 (−2.9, −0.2) *
$PNC_{1–2.5}$	−10.0 (−41.6, 21.7)	−3.0 (−26.8, 20.7)	0.1 (−0.3, 0.4)
$PNC_{2.5–10}$	−32.1 (−71.7, 7.5)	−4.9 (−32.4, 22.7)	0.1 (−0.2, 0.5)

Abbreviations: FEV_1, forced expiratory volume in 1 s; FVC, forced vital capacity; LVEF, left ventricular ejection fraction. Other abbreviations as in Table S1. * $p < 0.05$.
